# Vaccine‐preventable disease hospitalized patients with heart failure with reduced ejection fraction

**DOI:** 10.1002/clc.23800

**Published:** 2022-03-10

**Authors:** Juan Del Cid Fratti, Miguel Salazar, Erwin E. Argueta‐Sosa

**Affiliations:** ^1^ Cardiology Department, OSF Healthcare University of Illinois at Peoria Peoria Illinois USA; ^2^ Medicine Department, University Hospitals of Cleveland Medical Center Case Wester Reserve University Cleveland Ohio USA; ^3^ Cardiology Department Texas Tech University Health Science Lubbock Texas USA

**Keywords:** heart failure, influenza, pneumonia, vaccine, vaccine‐preventable disease

## Abstract

**Background:**

Over five million Americans suffer from heart failure (HF), and this is associated with multiple chronic comorbidities and recurrent decompensation. Currently, there is an increased incidence in vaccine‐preventable diseases (VPDs). We aim to investigate the impact of HF with reduced ejection fraction (HFrEF) in patients hospitalized with VPDs.

**Hypothesis:**

Patient with HFrEF are at higher risk for VPDs and they carry a higher risk for in‐hospital complications.

**Methods:**

Retrospective analysis from all hospital admissions from the 2016‐2018 National Inpatient Sample (NIS) using the ICD‐10CM codes for patients admitted with a primary diagnosis of VPDs with HFrEF and those without reduced ejection fraction. Outcomes evaluated were in‐hospital mortality, length of stay (LOS), healthcare utilization, frequency of admissions, and in‐hospital complications. Multivariate regression analysis was conducted to adjust for confounders.

**Results:**

Out of 317 670 VPDs discharges, we identified 12 130 (3.8%) patients with HFrEF as a comorbidity. The most common admission diagnosis for VPDs was influenza virus (IV) infection (75.0% vs. 64.1%; *p* < .01), followed by pneumococcal pneumonia (PNA) (13% vs. 9.4%; *p* < .01). After adjusting for confounders, patients with HFrEF had higher odds of having diagnosis of IV (adjusted [aOR], 1.42; *p* < .01) and PNA (aOR, 1.27; *p* < .01). Patients with VPDs and HFrEF had significantly higher odds of mortality (aOR, 1.76; *p* < .01), LOS, respiratory failure requiring mechanical ventilation, and mechanical ventilation for less than 96 h.

**Conclusion:**

Influenza and PNA were the most common VPDs admitted to the hospital in patients with a concomitant diagnosis of HFrEF. They were associated with increased mortality and in‐hospital complications.

**Abbreviations**
HAV: hepatitis A virus
HBV: hepatitis B virus
HF: heart failure
HFrEF: heart failure reduced ejection fraction
HZV: Herpes Zoster virus
IV: influenza virus
LOS: length of stay
PNA: pneumococcal pneumonia
VPD: vaccine‐preventable diseases
VZV: Varicella Zoster

## 1 BACKGROUND

Vaccines are considered one of the greatest achievements in medicine and have helped decrease the incidence and even eradicate several transmissible diseases. Vaccination contributes substantially to global health by preventing morbidity and mortality.^1^ Currently we are witnessing an increased incidence of VPDs in the United States which can be attributed to vaccine refusals, under‐vaccination, waning immunity, and migration from countries outside the United States.^2^, ^3^

As an example the overall vaccination coverage among adults in racial minorities is lower when compared with non‐Hispanic whites leading to racial and ethnic disparities.^4^ In the United States, HF is common; over 5 million Americans have this diagnosis with an annual incidence of 800 000 cases. Heart failure (HF)‐related hospitalizations are one of the most common reasons for admission in the United States, with more than one million hospitalizations annually for acute disease exacerbation. Patient outcomes remain poor, with an approximate 50% 5‐year survival rate, making this a crucial public health issue.^5^ HF has also been associated with inflammatory states with elevated serum proinflammatory cytokines. This suggests that diseases causing low‐grade chronic inflammation may be important contributors to HF progression.^6^ Thus infections can be an important cause of HF decompensation; these can be acquired in the community or during hospitalization, some leading to primary pulmonary infections.^7^ Vaccination in HF varies in the United States, and this patient population is more susceptible to influenza‐related complications like pneumonia, acute decompensation, and increased hospitalization complications. Data from a landmark clinical trial suggest that vaccination for influenza in HF was associated with a reduced risk of death.^8^

The aim of this study is to identify the outcomes of patients hospitalized with VPDs and a concomitant diagnosis of HFrEF.

## 2 METHODS

### 2.1 Study design and database description

The study was reviewed by the University of Illinois at Peoria Institutional Review Board, which exempted the study from institutional review board approval and waived the requirement of informed consent because the National Inpatient Sample (NIS) is a public, previously collected, and deidentified data. This is a retrospective cohort study of adult patients admitted with VPDs in acute‐care hospitals across the United States. Patients were selected from the NIS database. The database was created and is maintained by the Agency for Healthcare Research and Quality (AHRQ). It is the largest public inpatient database in the United States. It was designed as a stratified probability sample to be representative of all nonfederal acute care hospitals in the United States. Hospitals are stratified according to ownership/control, bed size, urban/rural location, teaching status, and geographical region. A 20% probability sample of all hospitals within each stratum is collected and then weighted to ensure that they are nationally representative. In 2016−2017, the NIS included 4575 hospitals in 47 states with 71 473 874 weighted discharges. The NIS sample contains patient data and hospital‐level information, which has been used to provide reliable estimates of cardiovascular disease burden. From January 1, 2016 to December 31, 2018, there were included 30 discharge diagnoses and 15 procedural diagnoses for each patient from the sample which can be identified using the ICD‐10‐CM/PCS codes.

### 2.2 Study patients

There is not a unique ICD‐10‐CM code for VPDs, but there are validated codes that may identify patients with these diseases. Only patients with principal ICD‐10‐CM diagnosis of VPDs from January 1, 2016 to December 31, 2018 were included in this study. The VPDs included in this study were pneumococcal pneumonia (PNA), Herpes Zoster virus (HZV), Varicella Zoster virus (VZV), meningococcal meningitis (MNC), influenza A virus, and other influenza type virus (IV), tetanus (TET), diphtheria (DIPTH), pertussis (WHC), acute Hepatitis A (HAV), acute Hepatitis B (HBV), rubella, measles and human papilloma virus (HPV). Patients were subdivided into the presence or absence of HFrEF if they had a secondary ICD‐10‐CM code for this diagnosis. Important patient comorbidities like diabetes mellitus (DM), history of organ transplant (OT), history of malignancy, and HIV infection were also identified with the ICD‐10‐CM discharge codes. The severity of comorbid conditions was defined using the modified Charlson's comorbidity index (CCI), which contains 17 weighted comorbid conditions with a score ranging from 0 to 33.^9^, ^10^ Patients younger than 18 years old were excluded. The specific ICD‐10‐CM codes that we used and the inclusion criteria flow chart are shown in Supporting Information Appendices C and D.

### 2.3 Study variables

Mortality, LOS, total hospitalization charges, and cost are provided within the NIS with each hospitalization. Potential confounders were gender, age, race, median yearly income per patient's zip code, CCI, hospital location (rural or urban), geographic region (Northeast, West, South, or Midwest), hospital teaching status, hospital beds, and individual patient comorbidities (OT, HIV, DM, or malignancy) that were identified with secondary ICD‐10‐CM.

### 2.4 Outcomes

The primary outcomes were proportions and admission odds for VPDs in patients with and without HFrEF. Secondary outcomes were in‐hospital mortality, healthcare utilization (total hospital charges and costs), length of stay provided by the NIS for each discharge, and in‐hospital complications (respiratory failure, mechanical ventilation for less than 24 h, for 24−96 h, and >96 h, acute kidney injury, shock, and sepsis) that were extracted with specific ICD‐10‐CM/PCS codes. We performed a secondary analysis for the primary and secondary outcomes independently for race and gender. Due to the low incidence of HPV, MNC, TET, DIPHT, and WHC, a variable called Rare VPDs was created with these discharge codes.

### 2.5 Statistical analysis

Analyses were performed using STATA version 16. The NIS is based on a complex sampling design that includes weighting, clustering, and stratification. STATA facilitates analysis to produce nationally representative unbiased results, variance estimates, and *p* values. We performed a multivariate logistic regression to adjust for confounding variables (diabetes mellitus, human immunodeficiency virus, history of organ transplant, malignancy, weekend admission, age, gender, race, income, CCI, hospital location, hospital region, teaching hospital, and hospital bed‐size). We included an extensive multivariate logistic regression analysis to adjust for covariate imbalance, selection bias, and potential confounders which we deem noninferior to a propensity score with covariate adjustment based on prior studies comparing both methods.^11^ Proportions were compared by using the Fischer exact test, and continuous variables were compared by using the Student's *t* test. All *p* values were two‐sided with a .05 as the threshold for statistical significance. The study adheres to best methodological practices for the NIS analysis to minimize the NIS limitations and to provide reliable results.^12^, ^13^

## 3 RESULTS

### 3.1 Patient characteristics

There were 107 001 355 discharges included in the NIS database in 2016−2018, of which 317  670 met the inclusion criteria. From this population, 12 130 (3.8%) patients had HFrEF as a comorbidity. When compared with patients without HFrEF, the HFrEF sample had a higher mean age (72 vs. 65 years; *p* < .01), were more likely to be males (59.4% vs. 43.7%; *p* < .01), African American (17% vs. 13%; *p* < .01), have higher CCI score ≥ 3 (74% vs. 30%; *p* < .01), were Medicare beneficiaries (77.2% vs. 61.1%; p < .01), have lower income per year (31.4% vs. 29.4; *p *= 0.03), resided in the Midwest (28.4% vs. 25.3%; *p* < .01), were admitted to large size (52.2% vs. 47.5%; *p* < .01), and teaching (68.6% vs. 63.9%; *p* < .01) hospitals. Patients with HFrEF are more likely to have DM (43.2% vs. 28.5%; *p* < .01), dyslipidemia (51.7% vs. 36.2%; *p* < .01), atrial fibrillation (44.4% vs. 16.1%; *p* < .01), chronic obstructive pulmonary disease (37% vs. 25.8%; *p* < .01), PAD (5.6% vs. 2.6%; *p* < .01), hypothyroidism (17.3% vs. 15.2%; *p* < .01), and chronic kidney disease (40.8% vs. 17.7%; *p* < .01) as comorbidities (Tables 1 and 2).

**Table 1:** Patient characteristics and hospital characteristics

	VPD discharges without HFrEF	VPD discharges with HFrEF	*p* value
Patient characteristics			
No. (%) of patients (317 670)	305 540	12 130	N/A
Female (%)	56.3	40.6	<.01
Mean age (years)	65.5	72.1	<.01
Weekend admission (%)	26.3	27.4	.21
Race (%)			
White	68.3	67.4	.38
African American	12.9	17.0	<.01
Hispanic	9.5	8.6	.14
Asian	2.7	2.1	.08
Native American	0.6	0.5	.67
Other	6.0	4.4	.01
Charlson Comorbidity Index score (%)			
0	22.52	0	<.01
1	28.6	8.12	<.01
2	18.8	17.8	.22
≥3	30.0	74.0	<.01
Median annual income in patient's zip code, US$ (%)			
1−42 999	29.4	31.4	.03
43 000−53 999	26.3	26.1	.81
54 000−70 999	23.3	22.7	.53
>71 000	19.3	18.3	.22
Insurance type, (%)			
Medicare	61.1	77.2	<.01
Medicaid	13.7	9.5	<.01
Private	18.3	9.7	<.01
Self‐pay	4.4	1.8	<.01
Hospital region (%)			
Northeast	20.7	21.5	.39
Midwest	25.3	28.4	<.01
South	36.2	33.4	<.01
West	17.7	16.8	.24
Hospital bed size (%)			
Small	23.7	20.4	<.01
Medium	28.8	27.5	.14
Large	47.5	52.2	<.01
Hospital location (%)			
Urban	87.5	89.6	<.01
Teaching hospital status (%)			
Teaching	63.9	68.6	<.01
Healthcare utilization resources			
Length of stay, mean (days)	4.1	5.0	<.01
Total charges, mean (US$)	34 033	43 100	<.01
Total costs, mean (US$)	8577	10 839	<.01

Abbreviations: HFrEF, heart failure and reduced ejection fraction; VPD, vaccine‐preventable diseases.

**Table 2:** Patient comorbidities, etiology of VPDs, and outcomes

	Patients without HFrEF	Patients with HFrEF	*p* value
Patient comorbidities (%)			
HIV infection	0.95	0.74	.28
Malignancy	11.3	12.2	.17
Alcohol abuse	3.9	3.5	.26
Tobacco use	1.3	1.2	.51
Cannabis use	2.1	1.1	<.01
Opioid abuse	3.0	1.3	<.01
T1DM	0.08	0.08	.87
T2DM	28.5	43.2	<.01
Cirrhosis	3.5	2.1	<.01
Dyslipidemia	36.2	51.7	<.01
Atrial fibrillation	16.1	44.4	<.01
COPD	25.8	37.0	<.01
PAD	2.6	5.6	<.01
HTN	41.5	10.6	<.01
Hypothyroid	15.2	17.3	<.01
CKD	17.7	40.8	<.01
Etiology of VPD (%)			
Influenza infection	64.1	75.0	<.01
Herpes Zoster	11.3	7.5	<.01
Varicella Zoster	0.8	0.4	.04
Hepatitis A	2.5	0.7	<.01
Hepatitis B	11.5	3.7	<.01
Pneumonia (Pneumococcal)	9.4	13.0	<.01
Bordetella Pertussis	0.1	0.08	.41
Diphtheria	0.05	0	.65
Meningococcal infection	0.2	0.2	.80
Tetanus	0.05	0.08	.61
Rare VPD	0.4	0.3	.54
Inpatient comorbidities (%)			
Respiratory failure/mechanical ventilation	1.5	3.2	<.01
Mechanical ventilation <24 h	0.3	0.8	<.01
Mechanical ventilation 24−96 h	0.6	1.6	<.01
Mechanical ventilation >96 h	0.5	0.9	<.01
AKI	13.6	22.7	<.01
Shock	0.2	0.6	<.01
Sepsis	1.3	1.1	.28
SNF transfer	17.0	22.7	<.01
HHC transfer	13.8	19.8	<.01
Died during hospitalization	0.9%	2.6%	<.01

Abbreviations: AKI, acute kidney injury; CKD, chronic kidney disease; COPD, chronic obstructive pulmonary disease; HFrEF, heart failure and reduced ejection fraction; HHC, home health care; HIV, human immunodeficiency virus; HTN, hypertension; PAD, peripheral arterial disease; SNF, skilled nursing facility; T1DM, Type 1 diabetes mellitus; T2DM, Type 2 Diabetes mellitus; VPD, vaccine‐preventable diseases.

### 3.2 VPD admission proportions in patients with HFrEF

**Figure 1:**
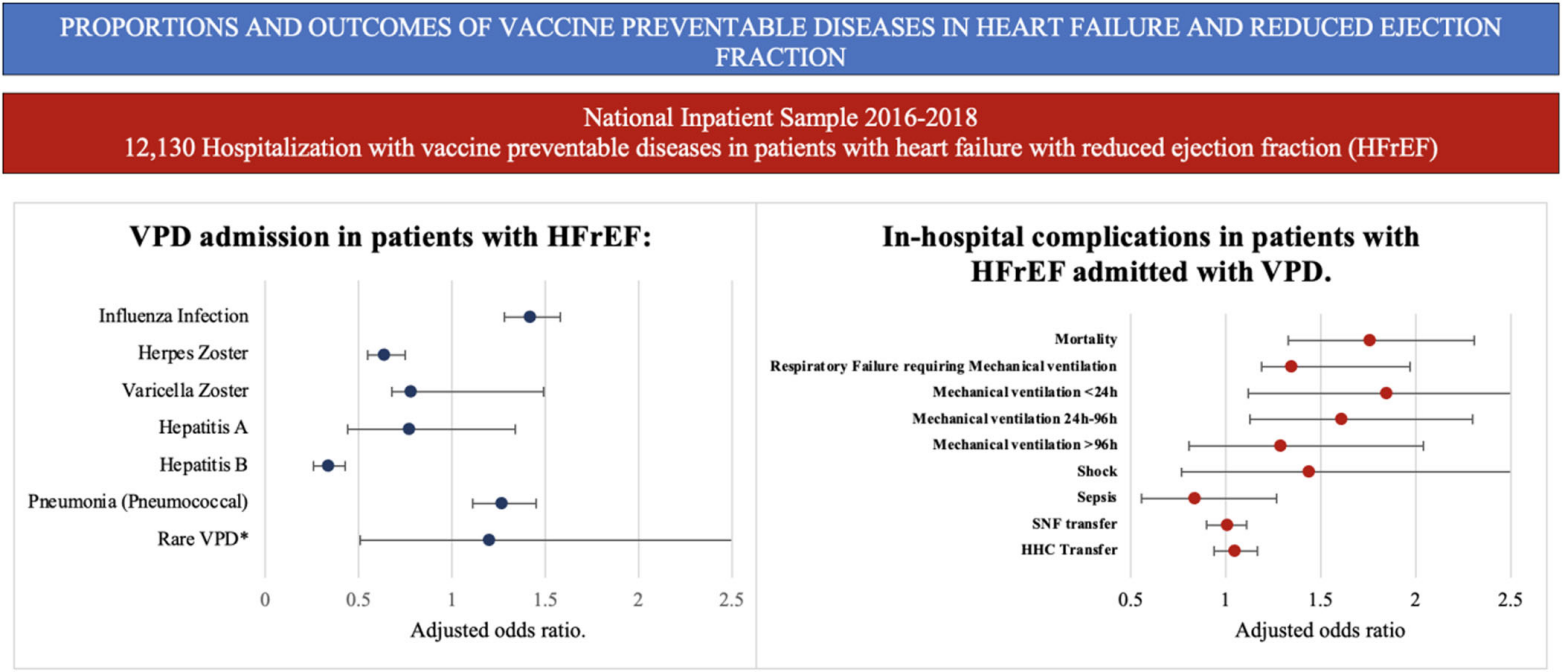
Proportions of admission and in‐hospital outcomes of vaccine‐preventable diseases in heart failure with reduced ejection fraction. *Pneumococcal proven pneumonia. **Rare VPD: HPV, MNC, TET, DIPHT, and WHC. DIPTH, diphtheria; HFrEF, heart failure and reduced ejection fraction; HPV, human papilloma virus; MNC, meningococcal meningitis; TET, tetanus; VPD, vaccine‐preventable diseases; WHC, pertussis

The proportion of VPDs in HFrEF when were IV 75%, HZV 7.5%, VZV 0.4%, HAV 0.7%, HBV 3.7%, PNA 13%, and Meningococcal infection 0.2%. No discharges from DIPHT, whooping cough and TET were reported. Multivariate regression analysis revealed that patients with HFrEF have higher odds of having IV as an admission diagnosis (adjusted OR, 1.42; *p* < .01), PNA (adjusted OR, 1.27; *p* < .01), and lower odds of HZV (adjusted OR, 0.64; *p* < .01) and HBV (adjusted OR, 0.34; *p* < .01). (Figure 1 and Table 3).

**Table 3:** Adjusted OR for VPDs admission in patients with HFrEF

VPD	Adjusted OR	*p* value
Reference: Patients without HFrEF		
Influenza infection	1.42 (1.28–1.58)	<.01
Herpes Zoster	0.64 (0.55–0.75)	<.01
Varicella Zoster	0.78 (0.68–1.49)	.46
Hepatitis A	0.77 (0.44–1.34)	.36
Hepatitis B	0.34 (0.26–0.43)	<.01
Pneumonia (Pneumococcal)	1.27 (1.11–1.45)	<.01
Rare VPD*	1.20 (0.51–2.83)	.66

Abbreviations: HFrEF, heart failure and reduced ejection fraction; OR, odds ratio; VPD, vaccine‐preventable diseases.

*Rare VPD: HPV, MNC, TET, DIPHT, and WHC.

### 3.3 In‐hospital mortality

The in‐hospital mortality proportion for patients with VPDs and HFrEF was 2.6% and 0.9% without HFrEF, respectively. On univariable analysis and multivariable analysis, HFrEF was an independent predictor of overall in‐hospital mortality compared with patients without HFrEF admitted with VPDs (adjusted OR, 1.76; *p* < .01) (Table 4).

**Table 4:** Adjusted OR for in‐hospital complications in patients with HFrEF admitted with VPD

	Adjusted OR	*p* value
Reference: Patients without HFrEF		
Mortality	1.76 (1.33–2.31)	<.01
Respiratory failure requiring mechanical ventilation	1.35 (1.19–1.97)	<.01
Mechanical ventilation <24 h	1.85 (1.12–3.03)	.01
Mechanical ventilation 24−96 h	1.61 (1.13–2.30)	<.01
Mechanical ventilation >96 h	1.29 (0.81–2.04)	.27
Need of tracheostomy	1.49 (0.52–4.28)	.44
AKI	1.07 (0.96–1.19)	.18
Shock	1.44 (0.77–2.67)	.24
Sepsis	0.84 (0.56–1.27)	.42
SNF transfer	1.01 (0.90–1.11)	.99
HHC transfer	1.05 (0.94–1.17)	.33

Abbreviations: AKI, acute kidney injury; HFrEF, heart failure and reduced ejection fraction; HHC, home health care; OR, odds ratio; SNF, skilled nursing facility; VPD, vaccine‐preventable diseases.

### 3.4 Hospital length of stay

The median LOS for patients with VPDs without HFrEF was 4.1 days and those with HFrEF was 5.0 days. After adjusting for confounders patients with HFrEF had higher odds of having a higher LOS (extra 0.31 days: *p* < .01). (Table 1 and Supporting Information Appendix A).

### 3.5 Total hospitalization charges/costs

The mean total hospitalization charges for patients with VPDs and HFrEF were $43 100 and $34 033 in patients without HFrEF, respectively. The mean total hospitalization cost for patients with VPDs and HFrEF was $11 291 and $8836 in patients without HFrEF, respectively. After adjusting for confounders there was no difference in charges and costs (Table 1 and Supporting Information Appendix A).

### 3.6 In‐hospital morbidity

Patients with HFrEF had a higher need for mechanical ventilation due to acute respiratory failure (adjusted OR, 1.35; *p* < .01), mechanical ventilation <24 h (adjusted OR, 1.85; *p *= 0.01), and mechanical ventilation 24−96 h (adjusted OR, 1.61; *p* < .01). No differences were found in other complications (Figure 1 and Table 4).

### 3.7 VPD outcomes: Gender and race based analysis

After adjusting for confounders, there were no significant differences in VPD, complications, hospital LOS, total charges, and total costs between genders and race (these findings are included in the Supporting Information Appendices A and B).

## 4 DISCUSSION

In this large national study with more than 317 540 VPD‐associated hospitalizations, we found that in patients with HFrEF, IV and PNA admissions are more common. HF‐related hospitalizations carry a great financial burden, can increase disease progression and death. Despite the decrease in the HF‐related admissions from 2006 to 2014, the disease burden is still high.^14^ With PNA being the second most common diagnosis in patients with HFrEF, preventing associated infections could lead to lower admissions for this group.^15^

HF has been recognized as a risk factor for worse outcomes in patients with seasonal influenza infection, with a high rate of acute decompensation, cardiovascular morbidity, and all‐cause mortality.^16^ A subgroup analysis from PARADIGM‐HF trial demonstrated that vaccination for influenza in patients with HFrEF was associated with a reduced risk of death, although this association is not well understood.^17^ A recent self‐controlled case series with a regression analysis done in Europe showed that in HF patients, influenza vaccination is associated with reduced risk of cardiovascular and all‐cause hospitalizations.^18^ In comparison, in our study by using national cohort data, we identified that patients admitted with VPDs and HFrEF had higher odds IV when compared with patients without HFrEF. VPD admissions and a concomitant diagnosis of HFrEF also carry a worse prognosis in terms of morbidity and mortality. Data from IAMI randomized trial demonstrated that influenza vaccination after acute myocardial infarction or high‐risk stable coronary artery disease patients decreased the risk of all‐cause mortality, cardiovascular mortality, myocardial infarction, and stent thrombosis at 12 months when compared with placebo that demonstrated the protective effect of influenza vaccination in high cardiovascular risk patients.^19^

Patients with HFrEF also had more odds of being admitted for PNA when compared with those without HFrEF. Prior data suggest that mortality is higher in this high‐risk cohort group.^20^ Also, there are studies that found that patients admitted for PNA have new or worsening HF at admission.^21^ Data from the Cardiovascular Health Study (CHS) collected from the National Heart, Lung and Blood Institute (NHLBI) have shown that the lack of pneumococcal vaccine in patients above 80 years of age is associated with an increased risk of mortality, incident HF, and higher pneumonia admission odds.^22^

In our study patients with HFrEF had a lower proportion of admission for HZV and HBV. A study cohort from Taiwan has shown that patients with HF may have an increased risk of Herpes Zoster^23^; the discrepancies of this study and our results are not clear, but it could be the difference in population, sample size, or the fact that in the Taiwanese study they used HF as a primary diagnosis and not Herpes Zoster. To our knowledge, there are no data about the incidence or outcomes of HBV infection in patients with HFrEF.

For the rest of the VPDs, there was no statistical difference in the proportions of admission when compared with patients without HFrEF. Regardless of these results, HF patients should continue to receive these vaccinations in accordance with CDC guidelines.

We found that in patients admitted with VPDs and a diagnosis of HFrEF, African Americans had a higher proportion when compared with other races. This could be explained due to higher comorbidities or lack of medical resources, after adjusting for confounders there was no difference admission for VPDs, data that differ from prior studies.^24^, ^25^ We did not identify a significant difference in admission for other VPDs or secondary outcomes by races.

Within the HFrEF group women had higher odds of admission with HZV. Prior studies have shown increased rates of shingles in unvaccinated older females.^26^ A metanalysis that evaluated risk factors for HZV infection showed that cardiovascular disease and female gender elevated the risk for HZV infection or reactivation.^27^

Another relevant point in VPD in HF is the surge of the Novel Coronavirus disease 2019 (COVID‐19) that in the past 2 years became the largest global health issue. Data from the CARD‐COVID program have demonstrated that patients with HF and COVID 19 are more prone to develop acute exacerbation and higher mortality during the course of the illness.^28^ One of the strongest predictor factors for mortality and complications after COVID19 is the prior history of HF.^29^ COVID‐19 vaccination has demonstrated to reduce the risk of infection, infection severity, complications, and mortality in all groups; for this reason, the European Society of Cardiology (ESC) and American College of Cardiology (ACC) recommended prioritizing vaccination in patients with HF without contraindications.^30^, ^31^

We demonstrated in our study that patients with HFrEF have higher proportions of being admitted for VPD and this carries worse in‐hospital outcomes.

## 5 LIMITATIONS

Our study is limited by being a retrospective analysis of the NIS, that relies on ICD‐10 codes for the population of interest, comorbidities/outcomes extraction; thus there could be variable misdiagnosis based on coding mistakes. It was also not possible to verify the vaccination status of the patient, or the type, duration, and severity of HFrEF. Patients with IV and PNA are more likely to be admitted to the hospital, and the rest of VPDs are usually managed as outpatient, and the NIS captures only inpatient records. This is an observational and retrospective study; there are potentials for selection bias and unmeasured confounders. In this study, we adhere to required practices for the NIS and performed an extensive multivariate regression analysis to try to mitigate these risks and deliver reliable results. We believe that this study reinforces the importance of vaccination, and the burden of HFrEF in this population.

## 6 CONCLUSION

This retrospective study demonstrates that from VPDs that require hospitalization, IV‐ and PNA‐related infections are more likely in patients with an HFrEF diagnosis and are associated with greater in‐hospital mortality and adverse clinical outcomes when compared with patients without HFrEF. Based on our sample African Americans are a population that may warrant additional studies to identify potential racial and ethnic disparities. Influenza and PNA vaccination in prior studies have been shown to reduce morbidity and mortality in patients with HF; our recent findings support why this could be significant. These results continue to emphasize why it can be important to address influenza and PNA vaccination in patients with HFrEF.

## CONFLICTS OF INTEREST

The authors declare no conflicts of interest.

## Supporting information

Supplementary information.

## Data Availability

Data are available upon request.
