# Supplementary material for: Vaccine‐preventable disease hospitalized patients with heart failure with reduced ejection fraction
Source: Clin Cardiol. 2022 Mar 10;45(5):474–81. doi: 10.1002/clc.23800 (PMC9045068; doi:10.1002/clc.23800)
Supplement: Supplementary file 1 — Supplementary information. [file CLC-45-474-s001.docx]

**APPENDIX A: Length of stay, charges, and cost analysis.**

| **Adjusted Differences for Secondary Outcomes in Patients with HFrEF Admitted with VPD.** | | | |
| --- | --- | --- | --- |
| **References: Patients without HFrEF.** | | | |
|  | **LOS** | **Total Charges** | **Total Cost** |
| VPD and HFrEF | Extra 0.31 days (0.12 – 0.51; p=<0.01) | Extra 1,338$ (Less 1,474 – Extra 4,141; p=0.35) | Extra 427$ (Less 248 – Extra 1,102; p=0.21) |

VPD= Vaccine preventable diseases; HFrEF= Heart failure and reduced ejection fraction; LOS= Length of stay.

**APPENDIX B: Gender and Race analysis:**

| **Adjusted Differences for VPD Admission by Race.** | | | | | | |
| --- | --- | --- | --- | --- | --- | --- |
| **Race** | **Influenza** | **HVZ** | **VZV** | **HAV** | **HBV** | **PNA** |
| Adjusted difference (P value) | | | | | | |
| **White** | Reference | | | | | |
| African American | 1.21 (p=0.19) | 1.22 (p=0.37) | 1.91 (p=0.40) | 0.53 (p=0.41) | 0.67 (p=0.24) | 0.73 (p=0.12) |
| Hispanic | 1.25 (p=0.22) | 1.23 (p=0.43) | N/A | N/A | 0.63 (0.32) | 0.76 (p=0.30) |
| Asian | 1.24 (p=0.56) | 0.71 (0.59) | N/A | N/A | 2.71 (p=0.15) | 0.45 (p=0.20) |

| **Adjusted Differences in Secondary Outcomes for VPD Admission by Race.** | | | | |
| --- | --- | --- | --- | --- |
| **Race** | **Mortality** | **LOS**  **(Days)** | **Total charges**  **(US$)** | **Total costs**  **(US$)** |
| Adjusted difference (P Value) | | | | |
| **White** | Reference | | | |
| African American | 1.46 (0.63 – 3.39; p=0.37) | Extra 0.79 days (Less 0.57 – Extra 0.72; p=<0.81) | Less 389$ (Less 9,453 – Extra 8,674; p=0.93) | Extra 75$ (Less 1,816 – Extra 1,966; p=0.93) |
| Hispanic | 1.29 (0.57 – 2.91; p=0.53) | Less 0.79 days (Less 1.3 to less 0.21 days; p=<0.01) | Less 1,531 (Less 10,263 – Extra 7,199; p=0.73) | Less 1,138$ (Less 3,020 – Extra 744; p=0.23) |
| Asian | 1.18 (0.28 – 4.93; p=0.28) | Less 0.89 days (Less 1.98 – extra 0.20; p=0.11) | Less 8,872$ (Less 22,995 – Extra 5,251; p=0.21) | Less 1,502$ (Less 5,168 – Extra 2,163; p=0.42) |
| Native American | N/A | Extra 0.80 (Less 1.37 – extra 2.97; p=0.47) | Extra 1,939$ (Less 20,408 – Extra 24,287; p=0.86) | Extra 2,002$ (Less 3,186 – 7,192; p=0.44) |

| **Crude Adjusted Differences for VPD Admission by gender.** | | | | | | |
| --- | --- | --- | --- | --- | --- | --- |
| **Gender** | **Influenza** | **HVZ** | **VZV** | **HAV** | **HBV** | **PNA** |
| Adjusted difference (P value) | | | | | | |
| **Male** | Reference | | | | | |
| Female | 1.11 (P:0.65) | 1.33 (P:0.04) | N/A | N/A | 0.72 (P:0.48) | 0.84 (P:0.84) |

| **Adjusted Differences for Secondary Outcomes in VPD Admission by Gender.** | | | | |
| --- | --- | --- | --- | --- |
| **Gender** | **Mortality** | **LOS**  **(Days)** | **Total charges**  **(US$)** | **Total costs**  **(US$)** |
| Adjusted difference (P Value) | | | | |
| Male | Reference | | | |
| Female | 0.92 (P:0.90) | 0.86 (P:0.20) | 22,667 (P:0.11) | 4,999 (P:0.16) |

**APPENDIX C: SUPPLEMENTAL DATA**

| ICD-10-CM/PCS codes used for analysis. | |
| --- | --- |
| Diagnosis | ICD-10-CM Code |
| **Heart Failure** | |
| Heart Failure with reduced EF | I502 |
| Unspecified systolic heart failure | I5020 |
| Acute systolic Heart Failure | I5021 |
| Chronic systolic heart failure | I5022 |
| Acute on Chronic Heart Failure | I5023 |
| VPDs | |
| Influenza | J0.9X1, J09X2, J09X9, J100, J101, J102, J108, J110, J111, J112, J118 |
| Herpes Zoster | B02 |
| Varicella Zoster | B01 |
| Hepatitis A | B15.0, B15.9 |
| Hepatitis B | B16, B17, B18, B19. |
| Pneumococcal Pneumonia | J13, J14, J15. |
| Bordetella Pertussis: | A37 |
| Diphtheria | A36 |
| Meningococcal Infection | A39 |
| Tetanus | A33, A34, A35 |
| Human Papilloma Virus | R87810, R87811, R87820, R87821 |
| **Comorbidities** | |
| HIV | B20 |
| Diabetes Mellitus | E08, E09, E10, E11, E13 |
| Malignancy | Z85 |
| Organ Transplant recipients | Z4821, Z4822, Z4823, Z4824, Z4828, Z4829 |
| **Complications** | |
| Acute Kidney Injury | N17, S37, N19 |
| Shock | R57, T811, T882 |
| Sepsis | R65 |
| Mechanical Ventilation | 5A09357, 5A09358, 5A09359, 5A0935B, 5A0935Z, 5A09457, 5A09458, 5A09459, 5A0945B, 5A0945Z, 5A09557, 5A09558, 5A09559, 5A0955B, 5A0955Z |
| Transfusion of Blood Products | 3023, 3024 |

**APPENDIX D. Inclusion criteria flow chart**


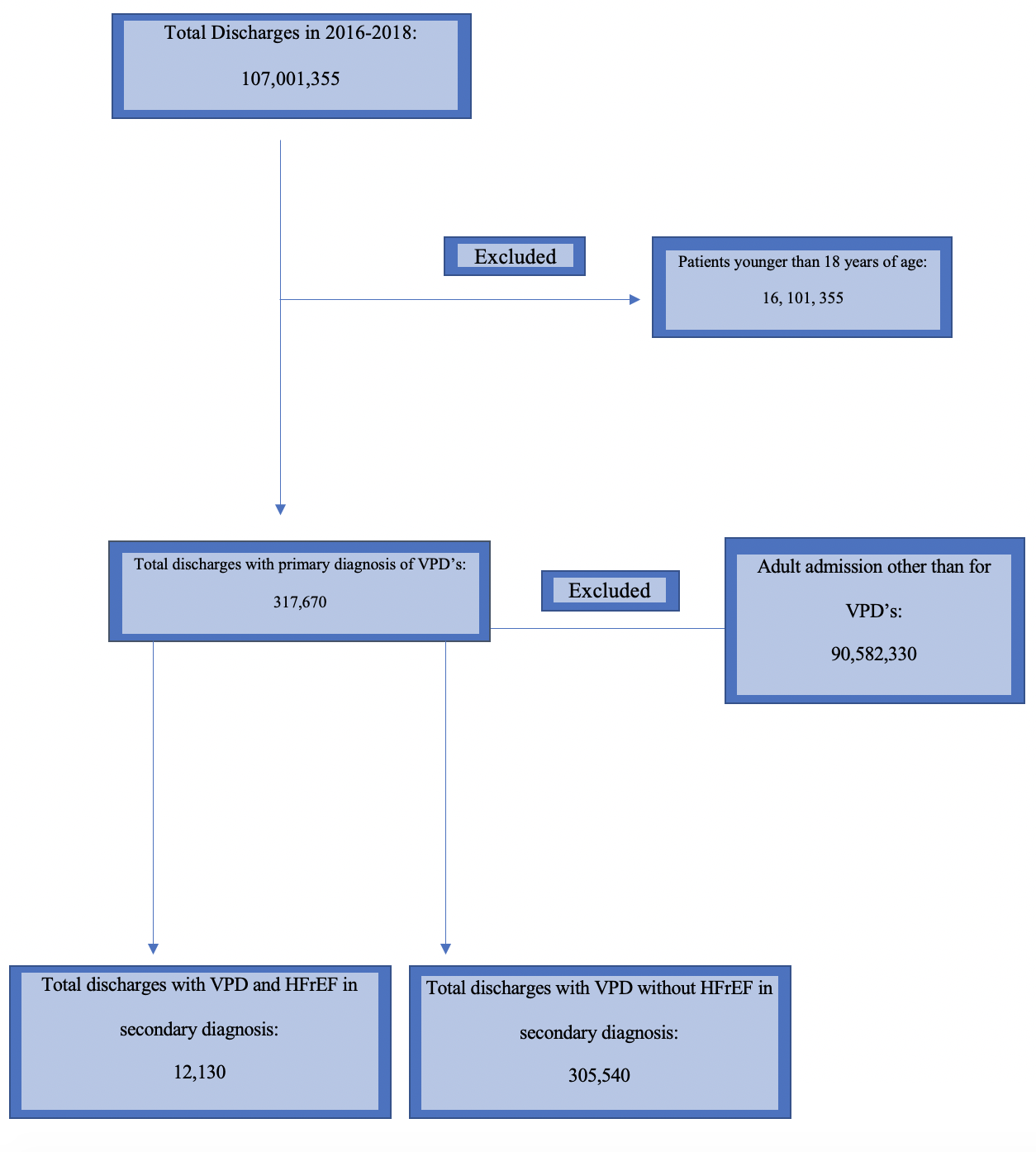


Inclusion criteria used in the study to obtain the sample.

VPD= Vaccine preventable disease; HFrEF: Heart failure and reduced ejection fraction.
